# Crystal structure of *N*-(3-benzoyl-4,5,6,7-tetra­hydro-1-benzo­thio­phen-2-yl)benzamide

**DOI:** 10.1107/S1600536814016948

**Published:** 2014-08-01

**Authors:** Manpreet Kaur, Jerry P. Jasinski, H. S. Yathirajan, Thammarse S. Yamuna, K. Byrappa

**Affiliations:** a Department of Studies in Chemistry, University of Mysore, Manasagangotri, Mysore 570 006, India; b Department of Chemistry, Keene State College, 229 Main Street, Keene, NH 03435-2001, USA; c Materials Science Center, University of Mysore, Vijyana Bhavan Building, Manasagangothri, Mysore-570 006, India

**Keywords:** crystal structure, hydrogen bonding, π–π stacking inter­actions, benzamide, 1-benzo­thio­phene, 2-amino­thio­phene derivatives

## Abstract

In the title compound, C_22_H_19_NO_2_S, the cyclo­hexene ring adopts a half-chair conformation. The dihedral angles between the plane of the thio­phene ring and those of its amide- and carbonyl-bonded benzene rings are 7.1 (1) and 59.0 (2)°, respectively. An intra­molecular N—H⋯O hydrogen bond generates an *S*(6) ring. In the crystal, very weak aromatic π–π stacking inter­actions [centroid–centroid separation = 3.9009 (10) Å] are observed.

## Related literature   

For applications of 2-amino­thio­phene derivatives, see: Sabnis *et al.* (1999 ▶); Puterová *et al.* (2010 ▶); Cannito *et al.* (1990 ▶); Nikolakopoulos *et al.* (2006 ▶); Lütjens *et al.* (2005 ▶). For a related structure, see: Kubicki *et al.* (2012 ▶).
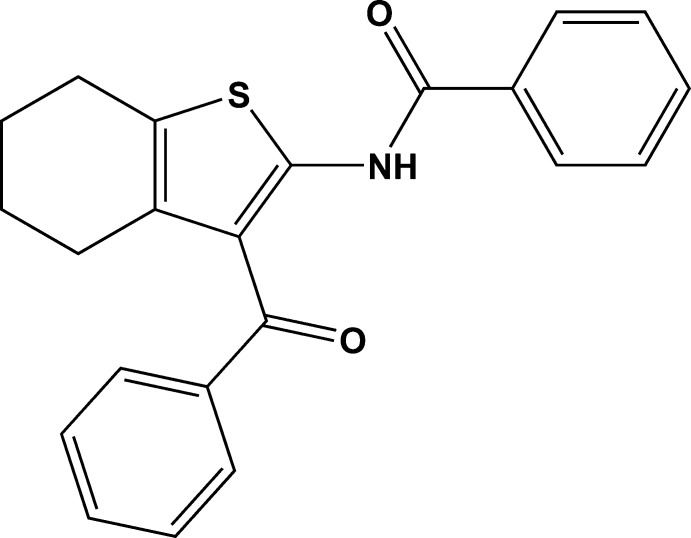


## Experimental   

### Crystal data  

C_22_H_19_NO_2_S
*M* *_r_* = 361.44
Monoclinic, 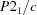

*a* = 13.5223 (4) Å
*b* = 6.23222 (15) Å
*c* = 22.2941 (6) Å
β = 106.150 (3)°
*V* = 1804.66 (9) Å^3^
*Z* = 4
Cu *K*α radiation
μ = 1.72 mm^−1^
*T* = 173 K
0.24 × 0.22 × 0.12 mm

### Data collection  

Agilent Eos Gemini diffractometer
Absorption correction: multi-scan (*CrysAlis RED*; Agilent, 2012 ▶) *T*_min_ = 0.698, *T*_max_ = 1.000
11346 measured reflections
3467 independent reflections
3049 reflections with *I* > 2σ(*I*)
*R*_int_ = 0.031

### Refinement  

*R*[*F* ^2^ > 2σ(*F* ^2^)] = 0.037
*wR*(*F* ^2^) = 0.105
*S* = 1.04
3467 reflections
235 parameters
H-atom parameters constrained
Δρ_max_ = 0.28 e Å^−3^
Δρ_min_ = −0.24 e Å^−3^

### 

Data collection: *CrysAlis PRO* (Agilent, 2012 ▶); cell refinement: *CrysAlis PRO*; data reduction: *CrysAlis RED* (Agilent, 2012 ▶); program(s) used to solve structure: *SUPERFLIP* (Palatinus & Chapuis, 2007 ▶); program(s) used to refine structure: *SHELXL2013* (Sheldrick, 2008 ▶); molecular graphics: *OLEX2* (Dolomanov *et al.*, 2009 ▶); software used to prepare material for publication: *OLEX2*.

## Supplementary Material

Crystal structure: contains datablock(s) I. DOI: 10.1107/S1600536814016948/hb7258sup1.cif

Structure factors: contains datablock(s) I. DOI: 10.1107/S1600536814016948/hb7258Isup2.hkl

Supporting information file. DOI: 10.1107/S1600536814016948/hb7258Isup3.cml

**Fig. 1.**
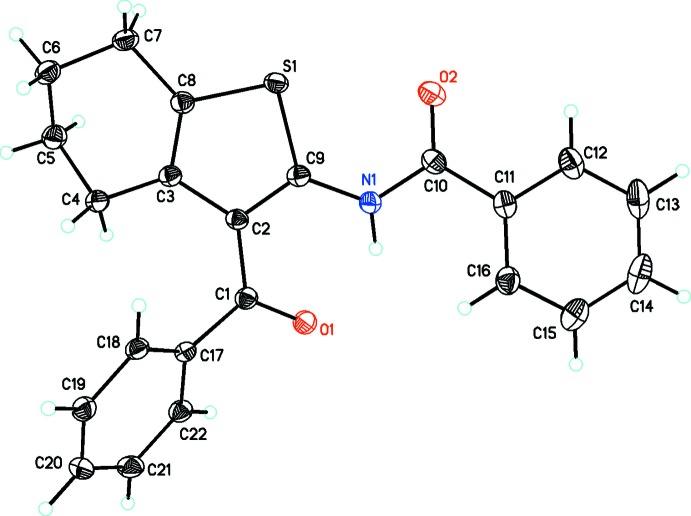
ORTEP drawing of C22H19NO2S showing 30% probability displacement ellipsoids.

22 19 2 . DOI: 10.1107/S1600536814016948/hb7258fig1.tif
ORTEP drawing of C_22_H_19_NO_2_S showing 30% probability displacement ellipsoids.

**Fig. 2.**
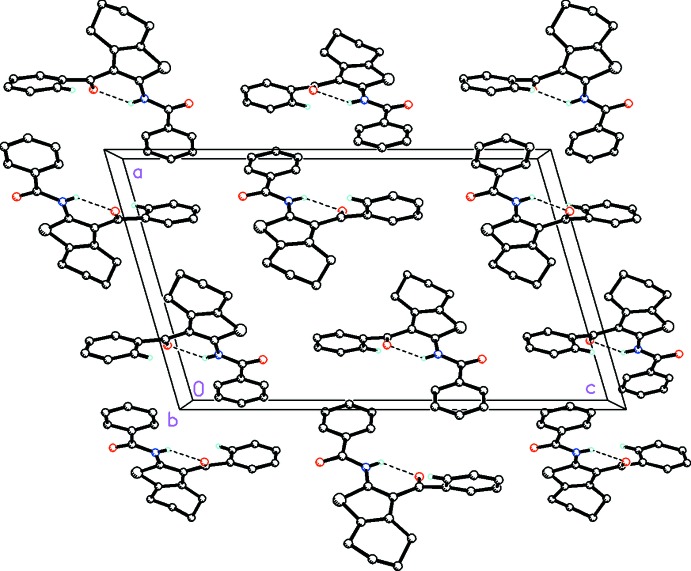
Molecular packing for C22H19NO2S viewed along the b axis. Dashed lines indicate N—H···O intramolecular hydrogen bonds. H atoms not involved in hydrogen bonding have been removed for clarity.

22 19 2 b . DOI: 10.1107/S1600536814016948/hb7258fig2.tif
Mol­ecular packing for C_22_H_19_NO_2_S viewed along the *b* axis. Dashed lines indicate N—H⋯O intra­molecular hydrogen bonds. H atoms not involved in hydrogen bonding have been removed for clarity.

CCDC reference: 1015542

Additional supporting information:  crystallographic information; 3D view; checkCIF report

## Figures and Tables

**Table 1:** Hydrogen-bond geometry (Å, °)

*D*—H⋯*A*	*D*—H	H⋯*A*	*D*⋯*A*	*D*—H⋯*A*
N1—H1⋯O1	0.86	2.01	2.6564 (16)	131

## supplementary crystallographic information

### S1. Structural commentary

2-Amino­thio­phene derivatives have been used in a number of applications in pesticides, dyes andpharmaceuticals. Reviews on the synthesis and properties of these compounds have been reported (Sabnis *et al.*, 1999; Puterová *et al.*, 2010). Substituted 2-amino­thio­phenes are active as allosteric enhancers at the human A1 adenosine receptor (Cannito *et al.*,1990; Nikolakopoulos *et al.*, 2006; Lütjens *et al.*, 2005). The crystal and molecular structures of two 2-amino­thiphenes have been previously reported by our group (Kubicki *et al.*, 2012). In continuation of our work on derivatives of 2-amino­thio­phenes, we report herin the crystal structure of the title compound, (I).

In (I), the cyclo­hexene ring adopts an envelope conformation (puckering parameters Q, θ, and φ = 0.5098 (19)Å, 126.7 (2)° and 322.2 (2)°, respectively) (Fig. 1). The dihedral angles between the mean planes of the thio­phene ring and phenyl rings are 7.1 (1)° and 59.0 (2)°. The phenyl rings are twisted with respect to each other by 54.1 (1)°. A short N1—H1···O1 intra­molecular hydrogen bond is observed. In addition, weak Cg–Cg π–π inter­molecular inter­actions are present (Cg1–Cg3 : 3.9009 (10)Å; x,1+y,z; Cg1: S1/C8/C3/C2/C9 and Cg3: C11–C16)(Fig 2).

### S2. Synthesis and crystallization

To a solution of benzoic acid (200 mg, 1.64 mmol) in di­chloro­methane (10 ml) was added 1-(3-di­methyl­amino­propyl)-3-ethyl­carbodimidide (377.26 mg, 1.968 mmol), tri­ethyl­amine (0.7 ml, 4.92 mmol) and stirred for 20 mins over a magnetic stirrer at room temperature. A solution of (2-Amino-4,5,6,7-tetra­hydro-benzo[b]thio­phen-3-yl)- phenyl-methanone (200 mg, 1.64 mmol) in 5 ml of di­chloro­methane was added to the above reaction mixture and continued stirring overnight at room temperature. The reaction completion was confirmed by thin layer chromatography. The reaction mixture was quenched with water and extracted with di­chloro­methane. The organic layers were separated, dried over anhydrous sodium sulphate and concentrated. The crude product was purified using silica gel column chromatography (60:120 mesh) using 20% ethyl­acetate in hexane. The column fractions for the title compound were left to evaporate in open air affording yellow blocks.

### S3. Refinement

Crystal data, data collection and structure refinement details are summarized in Table 1. All of the H atoms were placed in their calculated positions and then refined using the riding model with Atom—H lengths of 0.93Å (CH); 0.97Å (CH_2_) or 0.86Å (NH). Isotropic displacement parameters for these atoms were set to 1.2 (CH, CH_2_) times *U*_eq_ of the parent atom.

### Crystal data

**Table:**

C_22_H_19_NO_2_S	*F*(000) = 760
*M**_r_* = 361.44	*D*_x_ = 1.330 Mg m^−^^3^
Monoclinic, *P*2_1_/*c*	Cu *K*α radiation, λ = 1.54184 Å
*a* = 13.5223 (4) Å	Cell parameters from 5029 reflections
*b* = 6.23222 (15) Å	θ = 4.1–71.5°
*c* = 22.2941 (6) Å	µ = 1.72 mm^−^^1^
β = 106.150 (3)°	*T* = 173 K
*V* = 1804.66 (9) Å^3^	Rod, yellow
*Z* = 4	0.24 × 0.22 × 0.12 mm

### Data collection

**Table:**

Agilent Eos Gemini diffractometer	3467 independent reflections
Radiation source: Enhance (Cu) X-ray Source	3049 reflections with *I* > 2σ(*I*)
Detector resolution: 16.0416 pixels mm^-1^	*R*_int_ = 0.031
ω scans	θ_max_ = 71.2°, θ_min_ = 4.1°
Absorption correction: multi-scan (*CrysAlis RED*; Agilent, 2012)	*h* = −16→16
*T*_min_ = 0.698, *T*_max_ = 1.000	*k* = −7→7
11346 measured reflections	*l* = −18→26

### Refinement

**Table:**

Refinement on *F*^2^	Primary atom site location: structure-invariant direct methods
Least-squares matrix: full	Hydrogen site location: inferred from neighbouring sites
*R*[*F*^2^ > 2σ(*F*^2^)] = 0.037	H-atom parameters constrained
*wR*(*F*^2^) = 0.105	*w* = 1/[σ^2^(*F*_o_^2^) + (0.0622*P*)^2^ + 0.3587*P*] where *P* = (*F*_o_^2^ + 2*F*_c_^2^)/3
*S* = 1.04	(Δ/σ)_max_ = 0.001
3467 reflections	Δρ_max_ = 0.28 e Å^−^^3^
235 parameters	Δρ_min_ = −0.24 e Å^−^^3^
0 restraints	

### Special details

**Table:**

Experimental. 1H NMR (400 MHz, CDCl3): δ 12.60 (s, 1H), 8.04-8.02 (m, 2H), 7.57-7.54 (m, 3H), 7.52-7.42 (m, 5H), 2.72-2.69 (m, 2H), 1.96-1.93 (m, 2H), 1.79-1.76 (m, 2H), 1.54-1.51 (m, 2H). MS: m/z = 361.11 (Calculated), m/z = 361.977 [M]^+^ (found).
Geometry. All esds (except the esd in the dihedral angle between two l.s. planes) are estimated using the full covariance matrix. The cell esds are taken into account individually in the estimation of esds in distances, angles and torsion angles; correlations between esds in cell parameters are only used when they are defined by crystal symmetry. An approximate (isotropic) treatment of cell esds is used for estimating esds involving l.s. planes.

### Fractional atomic coordinates and isotropic or equivalent isotropic displacement parameters (Å^2^)

**Table:**

	*x*	*y*	*z*	*U*_iso_*/*U*_eq_	
S1	0.69329 (3)	0.62586 (6)	0.30922 (2)	0.02882 (13)	
O1	0.75720 (10)	0.27477 (18)	0.49345 (5)	0.0410 (3)	
O2	0.81305 (10)	0.2921 (2)	0.28522 (5)	0.0435 (3)	
N1	0.79387 (10)	0.2983 (2)	0.38260 (6)	0.0292 (3)	
H1	0.8088	0.2330	0.4180	0.035*	
C1	0.73063 (11)	0.4651 (2)	0.48717 (7)	0.0283 (3)	
C2	0.69969 (11)	0.5657 (2)	0.42510 (6)	0.0256 (3)	
C3	0.63392 (11)	0.7496 (2)	0.40467 (6)	0.0259 (3)	
C4	0.56991 (12)	0.8644 (3)	0.44032 (7)	0.0318 (3)	
H4A	0.6125	0.9671	0.4688	0.038*	
H4B	0.5438	0.7614	0.4648	0.038*	
C5	0.47979 (12)	0.9811 (3)	0.39537 (8)	0.0363 (4)	
H5A	0.4299	0.8768	0.3729	0.044*	
H5B	0.4462	1.0725	0.4190	0.044*	
C6	0.51630 (13)	1.1166 (3)	0.34902 (8)	0.0350 (4)	
H6A	0.5687	1.2161	0.3715	0.042*	
H6B	0.4590	1.1995	0.3238	0.042*	
C7	0.56026 (12)	0.9761 (3)	0.30670 (7)	0.0340 (3)	
H7A	0.5044	0.9144	0.2741	0.041*	
H7B	0.6020	1.0629	0.2870	0.041*	
C8	0.62504 (11)	0.7993 (2)	0.34390 (7)	0.0280 (3)	
C9	0.73396 (11)	0.4801 (2)	0.37706 (6)	0.0263 (3)	
C10	0.83182 (12)	0.2120 (2)	0.33703 (7)	0.0311 (3)	
C11	0.89548 (11)	0.0141 (2)	0.35530 (7)	0.0309 (3)	
C12	0.92489 (13)	−0.0932 (3)	0.30829 (9)	0.0428 (4)	
H12	0.9074	−0.0374	0.2680	0.051*	
C13	0.97989 (15)	−0.2821 (4)	0.32123 (11)	0.0562 (6)	
H13	0.9989	−0.3531	0.2895	0.067*	
C14	1.00688 (14)	−0.3666 (3)	0.38033 (12)	0.0549 (6)	
H14	1.0436	−0.4945	0.3886	0.066*	
C15	0.97916 (15)	−0.2603 (3)	0.42765 (10)	0.0485 (4)	
H15	0.9978	−0.3162	0.4679	0.058*	
C16	0.92373 (14)	−0.0708 (3)	0.41529 (8)	0.0391 (4)	
H16	0.9053	0.0000	0.4473	0.047*	
C17	0.73787 (11)	0.5924 (2)	0.54473 (6)	0.0259 (3)	
C18	0.77860 (11)	0.7983 (2)	0.55156 (7)	0.0278 (3)	
H18	0.7922	0.8668	0.5177	0.033*	
C19	0.79904 (12)	0.9025 (3)	0.60861 (7)	0.0332 (3)	
H19	0.8278	1.0392	0.6131	0.040*	
C20	0.77683 (13)	0.8037 (3)	0.65883 (7)	0.0375 (4)	
H20	0.7900	0.8742	0.6970	0.045*	
C21	0.73484 (13)	0.5988 (3)	0.65215 (7)	0.0382 (4)	
H21	0.7191	0.5331	0.6858	0.046*	
C22	0.71632 (12)	0.4922 (3)	0.59591 (7)	0.0314 (3)	
H22	0.6895	0.3539	0.5920	0.038*	

### Atomic displacement parameters (Å^2^)

**Table:**

	*U*^11^	*U*^22^	*U*^33^	*U*^12^	*U*^13^	*U*^23^
S1	0.0315 (2)	0.0324 (2)	0.02130 (19)	0.00008 (14)	0.00527 (14)	0.00048 (13)
O1	0.0672 (8)	0.0240 (6)	0.0324 (6)	0.0068 (5)	0.0152 (5)	0.0042 (4)
O2	0.0592 (8)	0.0429 (7)	0.0301 (6)	0.0045 (6)	0.0152 (5)	−0.0012 (5)
N1	0.0356 (7)	0.0257 (6)	0.0265 (6)	0.0014 (5)	0.0089 (5)	−0.0002 (5)
C1	0.0317 (7)	0.0247 (7)	0.0280 (7)	−0.0012 (6)	0.0077 (6)	0.0019 (6)
C2	0.0284 (7)	0.0245 (7)	0.0228 (7)	−0.0036 (6)	0.0052 (5)	−0.0009 (5)
C3	0.0260 (7)	0.0253 (7)	0.0245 (7)	−0.0029 (5)	0.0036 (5)	0.0004 (5)
C4	0.0330 (8)	0.0344 (8)	0.0276 (7)	0.0039 (6)	0.0082 (6)	0.0026 (6)
C5	0.0309 (8)	0.0421 (9)	0.0360 (8)	0.0062 (7)	0.0096 (6)	0.0036 (7)
C6	0.0343 (8)	0.0331 (8)	0.0339 (8)	0.0037 (6)	0.0032 (6)	0.0040 (6)
C7	0.0349 (8)	0.0372 (9)	0.0263 (7)	0.0035 (7)	0.0026 (6)	0.0058 (6)
C8	0.0263 (7)	0.0303 (8)	0.0254 (7)	−0.0011 (6)	0.0037 (6)	−0.0007 (6)
C9	0.0279 (7)	0.0244 (7)	0.0246 (7)	−0.0042 (6)	0.0040 (5)	−0.0003 (5)
C10	0.0333 (8)	0.0295 (8)	0.0305 (8)	−0.0066 (6)	0.0088 (6)	−0.0060 (6)
C11	0.0268 (7)	0.0290 (8)	0.0378 (8)	−0.0064 (6)	0.0104 (6)	−0.0081 (6)
C12	0.0352 (8)	0.0493 (10)	0.0452 (10)	−0.0036 (8)	0.0133 (7)	−0.0162 (8)
C13	0.0374 (10)	0.0572 (12)	0.0735 (14)	0.0054 (9)	0.0147 (9)	−0.0305 (11)
C14	0.0314 (9)	0.0367 (10)	0.0914 (16)	0.0037 (7)	0.0083 (10)	−0.0153 (10)
C15	0.0422 (10)	0.0364 (9)	0.0638 (12)	0.0037 (8)	0.0097 (9)	0.0046 (8)
C16	0.0431 (9)	0.0315 (8)	0.0439 (9)	0.0023 (7)	0.0141 (7)	−0.0011 (7)
C17	0.0257 (7)	0.0267 (7)	0.0238 (7)	0.0040 (6)	0.0046 (5)	0.0030 (5)
C18	0.0303 (7)	0.0265 (7)	0.0259 (7)	0.0025 (6)	0.0069 (6)	0.0055 (5)
C19	0.0329 (8)	0.0290 (8)	0.0330 (8)	0.0030 (6)	0.0012 (6)	−0.0018 (6)
C20	0.0409 (9)	0.0442 (9)	0.0240 (7)	0.0097 (7)	0.0033 (6)	−0.0038 (6)
C21	0.0432 (9)	0.0471 (10)	0.0265 (8)	0.0068 (7)	0.0134 (7)	0.0094 (7)
C22	0.0342 (8)	0.0303 (8)	0.0309 (8)	0.0017 (6)	0.0109 (6)	0.0070 (6)

### Geometric parameters (Å, º)

**Table:**

S1—C8	1.7363 (15)	C7—C8	1.505 (2)
S1—C9	1.7183 (14)	C10—C11	1.494 (2)
O1—C1	1.2359 (19)	C11—C12	1.391 (2)
O2—C10	1.219 (2)	C11—C16	1.389 (2)
N1—H1	0.8600	C12—H12	0.9300
N1—C9	1.3783 (19)	C12—C13	1.380 (3)
N1—C10	1.369 (2)	C13—H13	0.9300
C1—C2	1.4701 (19)	C13—C14	1.371 (3)
C1—C17	1.488 (2)	C14—H14	0.9300
C2—C3	1.444 (2)	C14—C15	1.383 (3)
C2—C9	1.387 (2)	C15—H15	0.9300
C3—C4	1.508 (2)	C15—C16	1.385 (2)
C3—C8	1.362 (2)	C16—H16	0.9300
C4—H4A	0.9700	C17—C18	1.388 (2)
C4—H4B	0.9700	C17—C22	1.401 (2)
C4—C5	1.529 (2)	C18—H18	0.9300
C5—H5A	0.9700	C18—C19	1.386 (2)
C5—H5B	0.9700	C19—H19	0.9300
C5—C6	1.519 (2)	C19—C20	1.382 (2)
C6—H6A	0.9700	C20—H20	0.9300
C6—H6B	0.9700	C20—C21	1.388 (3)
C6—C7	1.524 (2)	C21—H21	0.9300
C7—H7A	0.9700	C21—C22	1.379 (2)
C7—H7B	0.9700	C22—H22	0.9300
			
C9—S1—C8	90.96 (7)	N1—C9—C2	124.16 (13)
C9—N1—H1	117.0	C2—C9—S1	112.49 (11)
C10—N1—H1	117.0	O2—C10—N1	121.34 (15)
C10—N1—C9	126.06 (13)	O2—C10—C11	123.29 (14)
O1—C1—C2	120.92 (13)	N1—C10—C11	115.36 (13)
O1—C1—C17	117.80 (13)	C12—C11—C10	117.07 (15)
C2—C1—C17	121.15 (13)	C16—C11—C10	124.05 (14)
C3—C2—C1	128.64 (13)	C16—C11—C12	118.84 (16)
C9—C2—C1	119.60 (13)	C11—C12—H12	119.9
C9—C2—C3	111.75 (12)	C13—C12—C11	120.20 (19)
C2—C3—C4	127.10 (12)	C13—C12—H12	119.9
C8—C3—C2	111.73 (13)	C12—C13—H13	119.6
C8—C3—C4	120.77 (13)	C14—C13—C12	120.84 (18)
C3—C4—H4A	109.6	C14—C13—H13	119.6
C3—C4—H4B	109.6	C13—C14—H14	120.2
C3—C4—C5	110.47 (12)	C13—C14—C15	119.55 (18)
H4A—C4—H4B	108.1	C15—C14—H14	120.2
C5—C4—H4A	109.6	C14—C15—H15	119.9
C5—C4—H4B	109.6	C14—C15—C16	120.2 (2)
C4—C5—H5A	109.4	C16—C15—H15	119.9
C4—C5—H5B	109.4	C11—C16—H16	119.8
H5A—C5—H5B	108.0	C15—C16—C11	120.34 (17)
C6—C5—C4	111.07 (13)	C15—C16—H16	119.8
C6—C5—H5A	109.4	C18—C17—C1	121.09 (13)
C6—C5—H5B	109.4	C18—C17—C22	119.26 (13)
C5—C6—H6A	109.4	C22—C17—C1	119.09 (13)
C5—C6—H6B	109.4	C17—C18—H18	119.8
C5—C6—C7	111.01 (13)	C19—C18—C17	120.38 (14)
H6A—C6—H6B	108.0	C19—C18—H18	119.8
C7—C6—H6A	109.4	C18—C19—H19	119.9
C7—C6—H6B	109.4	C20—C19—C18	120.12 (15)
C6—C7—H7A	109.6	C20—C19—H19	119.9
C6—C7—H7B	109.6	C19—C20—H20	120.1
H7A—C7—H7B	108.1	C19—C20—C21	119.85 (15)
C8—C7—C6	110.29 (12)	C21—C20—H20	120.1
C8—C7—H7A	109.6	C20—C21—H21	119.8
C8—C7—H7B	109.6	C22—C21—C20	120.39 (15)
C3—C8—S1	113.01 (11)	C22—C21—H21	119.8
C3—C8—C7	126.25 (14)	C17—C22—H22	120.0
C7—C8—S1	120.68 (11)	C21—C22—C17	119.98 (15)
N1—C9—S1	123.35 (11)	C21—C22—H22	120.0
			
O1—C1—C2—C3	155.63 (15)	C8—S1—C9—N1	177.76 (13)
O1—C1—C2—C9	−23.4 (2)	C8—S1—C9—C2	−2.17 (12)
O1—C1—C17—C18	135.47 (15)	C8—C3—C4—C5	16.3 (2)
O1—C1—C17—C22	−35.8 (2)	C9—S1—C8—C3	0.70 (12)
O2—C10—C11—C12	−6.8 (2)	C9—S1—C8—C7	−176.53 (13)
O2—C10—C11—C16	175.24 (16)	C9—N1—C10—O2	−1.2 (2)
N1—C10—C11—C12	172.35 (14)	C9—N1—C10—C11	179.62 (13)
N1—C10—C11—C16	−5.6 (2)	C9—C2—C3—C4	170.22 (14)
C1—C2—C3—C4	−8.8 (2)	C9—C2—C3—C8	−2.51 (18)
C1—C2—C3—C8	178.44 (14)	C10—N1—C9—S1	2.1 (2)
C1—C2—C9—S1	−177.81 (10)	C10—N1—C9—C2	−177.95 (14)
C1—C2—C9—N1	2.3 (2)	C10—C11—C12—C13	−177.16 (16)
C1—C17—C18—C19	−170.37 (14)	C10—C11—C16—C15	177.18 (16)
C1—C17—C22—C21	171.94 (14)	C11—C12—C13—C14	−0.3 (3)
C2—C1—C17—C18	−40.4 (2)	C12—C11—C16—C15	−0.7 (3)
C2—C1—C17—C22	148.30 (14)	C12—C13—C14—C15	−0.4 (3)
C2—C3—C4—C5	−155.81 (14)	C13—C14—C15—C16	0.6 (3)
C2—C3—C8—S1	0.89 (16)	C14—C15—C16—C11	0.0 (3)
C2—C3—C8—C7	177.93 (14)	C16—C11—C12—C13	0.9 (3)
C3—C2—C9—S1	3.05 (16)	C17—C1—C2—C3	−28.6 (2)
C3—C2—C9—N1	−176.88 (13)	C17—C1—C2—C9	152.41 (14)
C3—C4—C5—C6	−49.88 (18)	C17—C18—C19—C20	−1.4 (2)
C4—C3—C8—S1	−172.37 (11)	C18—C17—C22—C21	0.5 (2)
C4—C3—C8—C7	4.7 (2)	C18—C19—C20—C21	0.6 (2)
C4—C5—C6—C7	64.62 (18)	C19—C20—C21—C22	0.8 (3)
C5—C6—C7—C8	−41.43 (18)	C20—C21—C22—C17	−1.4 (2)
C6—C7—C8—S1	−175.02 (11)	C22—C17—C18—C19	0.9 (2)
C6—C7—C8—C3	8.1 (2)		

### Hydrogen-bond geometry (Å, º)

**Table:**

*D*—H···*A*	*D*—H	H···*A*	*D*···*A*	*D*—H···*A*
N1—H1···O1	0.86	2.01	2.6564 (16)	131
