# Supplementary figures and images for: Crystal structure of N-(3-benzoyl-4,5,6,7-tetra­hydro-1-benzo­thio­phen-2-yl)benzamide
Source: Acta Crystallogr Sect E Struct Rep Online. 2014 Aug 1;70(Pt 9):o951–2. doi: 10.1107/S1600536814016948 (PMC4186148; doi:10.1107/S1600536814016948)

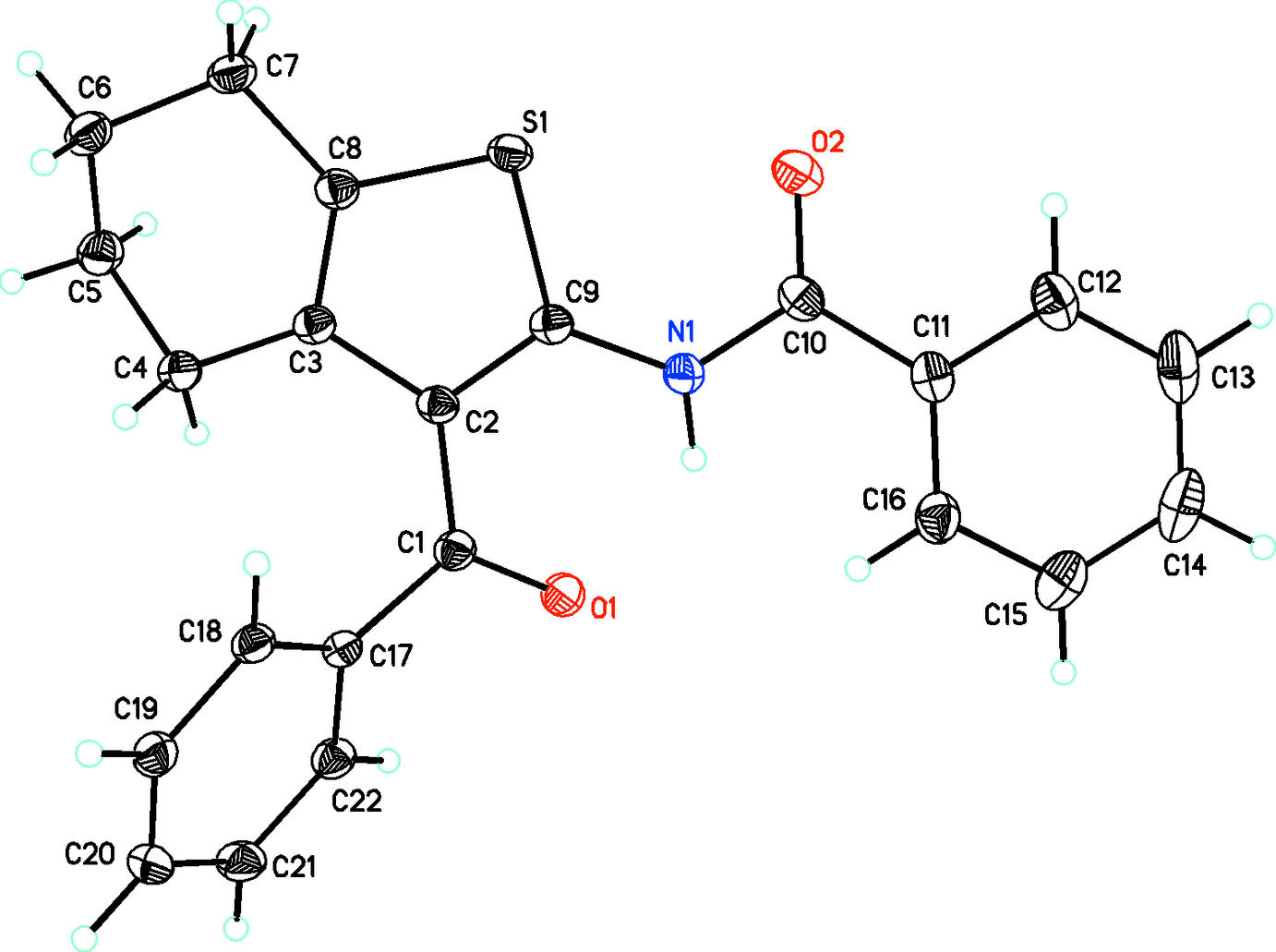

Supplement: Supplementary file 4 [file e-70-0o951-fig1.tif]

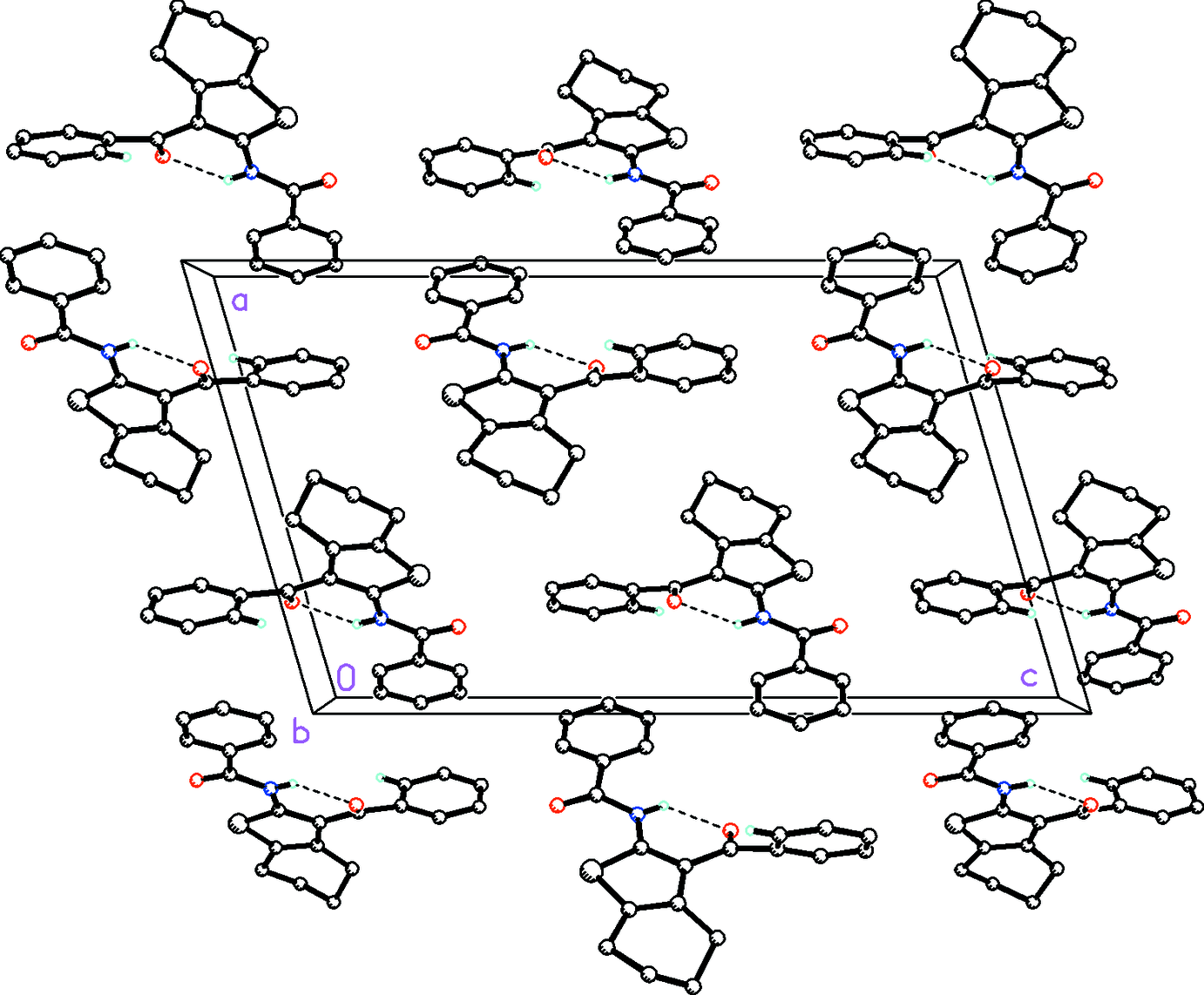

Supplement: Supplementary file 5 [file e-70-0o951-fig2.tif]
